# Appearance of β-lactam Resistance Genes in Agricultural Soils and Clinical Isolates over the 20^th^ Century

**DOI:** 10.1038/srep21550

**Published:** 2016-02-16

**Authors:** David W. Graham, Charles W. Knapp, Bent T. Christensen, Seánín McCluskey, Jan Dolfing

**Affiliations:** 1 School of Civil Engineering & Geosciences, Newcastle University, Newcastle upon Tyne, United Kingdom, NE1 7RU; 2 Department of Civil and Environmental Engineering, University of Strathclyde, Glasgow, United Kingdom, G1 1XJ; 3 Department of Agroecology, Aarhus University, AU-Foulum, DK-8830 Tjele, Denmark

## Abstract

Debate exists about whether agricultural versus medical antibiotic use drives increasing antibiotic resistance (AR) across nature. Both sectors have been inconsistent at antibiotic stewardship, but it is unclear which sector has most influenced acquired AR on broad scales. Using qPCR and soils archived since 1923 at Askov Experimental Station in Denmark, we quantified four broad-spectrum β-lactam AR genes (ARG; *bla*_TEM_, *bla*_SHV_, *bla*_OXA_ and *bla*_CTX-M_) and class-1 integron genes (*int*1) in soils from manured (M) versus inorganic fertilised (IF) fields. “Total” β-lactam ARG levels were significantly higher in M versus IF in soils post-1940 (paired-t test; p < 0.001). However, dominant individual ARGs varied over time; *bla*_TEM_ and *bla*_SHV_ between 1963 and 1974, *bla*_OXA_ slightly later, and *bla*_CTX-M_ since 1988. These dates roughly parallel first reporting of these genes in clinical isolates, suggesting ARGs in animal manure and humans are historically interconnected. Archive data further show when non-therapeutic antibiotic use was banned in Denmark, *bla*_CTX-M_ levels declined in M soils, suggesting accumulated soil ARGs can be reduced by prudent antibiotic stewardship. Conversely, *int*1 levels have continued to increase in M soils since 1990, implying direct manure application to soils should be scrutinized as part of future stewardship programs.

Mass use of antibiotics for treating infectious disease over the 20^th^ century has improved human health and agricultural productivity in the developed world, enhancing the quality of life of millions^1^. However, antibiotic use, especially in medicine and agriculture, also has fuelled increasing acquired antibiotic resistance (AR) in exposed organisms to a point where many antibiotics are compromised; multi-resistance pathogens are common; and new antibiotic development has become uneconomical^2^,^3^,^4^. Despite growing awareness of the AR crisis (e.g., ref. 5), debate continues over who and which activities are most responsible for increased AR in nature, largely because it is hard to link specific causative actions with explicit AR consequences^6^,^7^. AR is ancient with VanX proto-resistance and multi-resistance genes being detected in ~30,000-year old DNA from permafrost^8^. However, increased antibiotic use has clearly mobilised AR genes and accelerated bacterial AR evolution in strains not previously intrinsically resistant^9^,^10^, including human pathogens^11^. Antibiotic overuse and poor water quality in some parts of the world have further altered the environmental resistome (i.e., the pool of all AR genes; ARG)^12^,^13^, which increases the probability of AR acquisition in any exposed bacteria^14^.

Despite the above, debate continues about the relative role of each sectoral driver of acquired AR: medicine, agriculture or environmental pollution. This debate often follows parochial bias, but it also stems from substantial difficulties in identifying root causes of detected AR in most scenarios. AR often results from acquisition or mutation of a defence gene, which allows an organism to better protect itself. However, phenotypic detection of AR is only observed when a strain is exposed to an antibiotic. Therefore, organisms might acquire the potential for AR (e.g., an ARG or mutation) in one place, but phenotypic AR only becomes apparent when the “next” antibiotic treatment fails, possibly great distances away^15^. This reality has led many to falsely presume AR acquisition is primarily driven by factors at the point of detection (e.g., a hospital) when, in fact, original acquisition of ARGs or mutations might occur elsewhere, including the natural environment.

Within this context, long-term soil archives from the Netherlands were studied to assess relative ARG abundances in soils harvested since large-scale antibiotic manufacture increased in the 1940s^16^. This work showed relative soil ARG abundances, based on extracted DNA, have increased over the last 60 years. Although interesting, this work did not identify specific local causes for increasing ARG levels because detailed soil histories were not available. However, systematic soil archiving has been performed since 1923 at The Askov Long-Term Experiment (LTE) Station in Denmark, initiated in 1894 to study the role of animal manure versus inorganic fertilisers on soil fertility^17^. Detailed soil and field management records were kept for the Askov-LTE, which included fields that only received inorganic fertilizers (IF) over their entire history, whereas other fields received only animal manure (M). As such, the Askov-LTE soil archive provides a wholly unique platform for assessing long-term changes in *in situ* ARGs, especially relative impacts of animal versus non-animal factors on soil AR.

Here, DNA was harvested from archived Askov-LTE soils collected from 1923 to 2010, and relative abundances of four β-lactam ARGs (*bla*_TEM_, *bla*_SHV_, *bla*_OXA_ and *bla*_CTX-M_) and class 1 integron genes (*int*1) were contrasted over time. These ARGs, which code for broad (*bla*_TEM_ and *bla*_SHV_) and extended-spectrum (*bla*_OXA_ and *bla*_CTX-M_) β-lactamases (ESBL), were chosen as “biomarkers” for the appearance of β-lactam resistance over time because they have distinct histories in hospitals between 1963 and 1989^18^,^19^,^20^,^21^, and can confer resistance against an essential class of antibiotics for the treatment of infectious disease. With these data, one can contrast soil ARG levels associated with long-term fertiliser use and parallel observations in medicine, and also assess whether changes in Danish agricultural antibiotics practice has influenced relative ARG abundances in associated soils.

## Materials and Methods

### Field Experiment and Soil Archive

Soils assessed in this study were retrieved from the soil archive of the Askov-LTE initiated in 1894 at the Lermarken site^17^. The experimental site is located in South Jutland, Denmark and was first cultivated around 1800. It has mean annual temperature and precipitation of 7.7 °C and 862 mm, respectively, and is flat (gradient <2%) and well-sheltered by hedgerows and scattered woodlands. The soils are dominated by glacial moraine deposits, and Ap-horizons (0–18/20 cm) are characterized as coarse sandy loam.

Field experiments with different fertilization treatments have been performed on four sets of fields at the Lermarken site since 1894^17^,^22^. Field treatments have included different levels (½x, 1x, 1½x, 2x) of animal manure, inorganic fertilisers only, and unamended plots (no manure or inorganic fertilisers). Absolute nutrient amendments were increased in 1923 and again in 1973 (to align with mainstream fertilization levels in Danish agriculture), but almost identical amounts of N, P, and K were applied to both M and IF fields throughout the experiment. The Askov-LTE experiment assessed classical four-course crop rotation, including winter cereals, row crops, spring cereals and grass-clover mixture. Typically, fertilization has been performed in late autumn to early spring; soils for archiving were sampled during the same period, but always before next fertilization. Soil samples have been collected from the Ap-horizon every four years since 1923, air dried, and archived at room temperature under dry conditions, creating one of the longest agricultural soil archives in Europe.

For this study, archived soil samples were only quantified from 1× IF and 1× M fields between 1923 and 2010 to ensure consistency in field amendments. Actual IF and M treatments were 70 kg total-N, 16–19 kg P and 58–70 kg K between 1923 and 1973, and 100 kg total-N ha^−1^, 19 kg P ha^−1^ and 87 kg K ha^−1^ since 1973. Further details are found in Christensen *et al.*^17^,^22^.

### Chemical Soil Analyses

Total-C (TC) and -N (TN) in the soils were analysed by dry combustion and Kjeldahl digestion, respectively^22^. Extractable inorganic P (Ext P) was determined by extracting soil in 0.2 N sulphuric acid, whereas extractable K (Ext K) was extracted in 0.5 M ammonium-acetate^17^. Concentrations of heavy metals and trace elements were determined using ICP-MS following Aqua-Regia digestion.

### DNA Extraction and Gene Quantification

DNA was extracted using the FastDNA SPIN (MP Biomedical, Cambridge, UK) columns for soils, following manufacturer’s protocols. Typically, 200–300 mg (as dry weight) of dried soil was aseptically transferred into centrifuge tubes containing phosphate buffer saline (PBS) and extraction beads (weighing tubes before and after soil addition). Samples were gently mixed in the PBS for 15–20 minutes for rehydration, and cells were then lysed using a FastPrep cell disruptor (6.0 setting, 30 seconds; MP Biomedicals). Cell lysis was repeated twice. Resultant DNA was temporarily stored at −20 °C until all soils were extracted, and then retained at −80 °C prior to qPCR analysis.

Four β-lactam ARGs (*bla*_TEM_, *bla*_SHV_, *bla*_OXA_ and *bla*_CTX-M_) were chosen for quantification over time, which was based on previous experience assessing soils and sediments^16^,^23^,^24^,^25^ and their relative importance to therapeutic applications^3^. Primers for *bla*_TEM_^26^, *bla*_SHV_^27^,^28^, *bla*_CTX-M_^29^ and *bla*_OXA_^30^,^31^ target conservative regions among common β-lactamase genes (which encode for enzymes that inactivate penicillin and other β-lactam antibiotics). *Int*1 and 16S-rRNA bacterial gene abundances were quantified according to methods from Mazel *et al.*^32^ and Yu *et al.*^33^, respectively.

For qPCR analysis, two μl of DNA template and appropriate primers were combined with sso-Fast EvaGreen PCR reagent (Bio-Rad, Hercules, CA, USA) and molecular-grade water to create 20-μl reaction volumes. Analyses were performed using a Bio-Rad CFX96 system. Temperature cycles were 95 °C (30 sec) for enzyme activation, and then 40 cycles of 94 °C (5 sec), annealing temperatures (*bla*_TEM_: 50 °C, and 55 °C for *bla*_SHV_, *bla*_CTX-M_ and *bla*_OXA_) for 10 seconds, and 72 °C for an additional 5 seconds. Samples were analysed in duplicate; any samples with a major discrepancy (high analytical variability) were re-analysed. Duplicate cycle-threshold values always ranged within ±0.3 units (*log* scale). A post-analytical temperature melt curve was used to verify reaction quality (50–95 °C, ΔT = 0.1 °C/second).

All reactions were run with serially diluted plasmid-DNA standards derived from gene-positive bacteria. qPCR reaction efficiencies were determined by spiking sample with known amounts of DNA template, and results were compared with “neat” standards. Samples were typically diluted, either 1:100 or 1:1000, to reduce possible inhibitory effects from substances that co-elute when extracting soil DNA. Correlation coefficients for all standard curves were r > 0.98; and log gene-abundance values (except those below detection limits) were within the linear range of the calibration curves.

### Data Processing and Analyses

ARG abundances were normalised to 16S-rRNA gene abundances to minimise variance caused by differential extraction and analytical efficiencies, and differences in background bacterial abundances. Two-sample tests were then employed to statistically compare different groupings of the normalised data (e.g., M vs IF fields), including the t-test for normally-distributed datasets or the Wilcoxon Ranked-Sum test for non-normally-distributed datasets. Bivariate correlation analyses across samples used the Spearman rank method because many of the variables were non-normally-distributed, even after log-transformation.

To better visualize gene-abundance changes over time, all relative gene abundances (i.e., normalized to 16S gene values) were further normalized relative to mean ARG levels detected in soils from 1923 and 1938, which represent a timeframe before mass-production of antibiotics commenced. To validate this assumption, ARG data were statistically compared between M and IF soils archived from 1923 and 1938, and no significant differences were observed between field treatments. Therefore, data from are 1923 and 1938 samples were combined to quantify pre-1940 baseline levels of ARGs (and heavy metals and nutrients). Unless otherwise noted, significance is defined as 95% confidence (i.e., p < 0.05). All statistics were conducted using SPSS^TM^ version 22.

## Results and Discussion

Antibiotics have been used in medicine and agriculture since the 1930s. Initially, antibiotics only were used for medical applications, but their use expanded to agriculture in the 1950s, including therapeutic and non-therapeutic applications^2^. As part of this trend, Denmark was among the leaders in employing antibiotics to increase agricultural productivity and industrial animal production, including non-therapeutic use (e.g., growth promotion)^34^. However, in the 1990s, antibiotics were banned for non-therapeutic use and were slowly phased out^35^,^36^, and Denmark has become a benchmark for prudent antibiotic stewardship. As such, the Danish experience provides an ideal template for studying the historic effects of antibiotic use, including pre-antibiotic conditions, large-scale antibiotic use in agriculture, and conditions after antibiotics were banned for non-therapeutic use since the 1990s.

Previous work^16^ showed that relative levels of ARGs in Dutch agricultural soils significantly increased between the 1940s and 2010. For example, selected broad-spectrum β-lactam ARG genes (*bla*_TEM_ and *bla*_SHV_) increased by 15 times over this time, especially since the 1980s. Unfortunately, field data (e.g., type and amounts of fertilisers), irrigation sources, and other management factors varied across the Dutch study sites and soils, which made it difficult to determine specific field-level cause-and-effects relative to increased ARGs in the soils. Therefore, this earlier work was incomplete and more work was needed.

Here we used similar processing and ARG quantification methods as the Dutch study, but on soil archives with well-characterised field-management histories, particularly associated with M versus IF fertilization for over 100 years. Associated with M and IF fields, we quantified the selected *bla* ARGs, *int*1 and 16S rRNA gene abundances in archived soils since 1923. TC, TN, ExtP, ExtK, and selected heavy metal levels also were quantified over time to compare soil conditions with the ARG and *int*1 data. *int*1 levels were also quantified here because they are sometimes linked with acquired AR, especially due to animal waste releases^10^, and can imply an increased potential for horizontal gene transfer (HGT) in any ecosystem. As previously noted in the Methods, all post-1940 genetic and chemical data were normalised to pre-1940 values from 1923 and 1938 samples.

**Figure 1:**
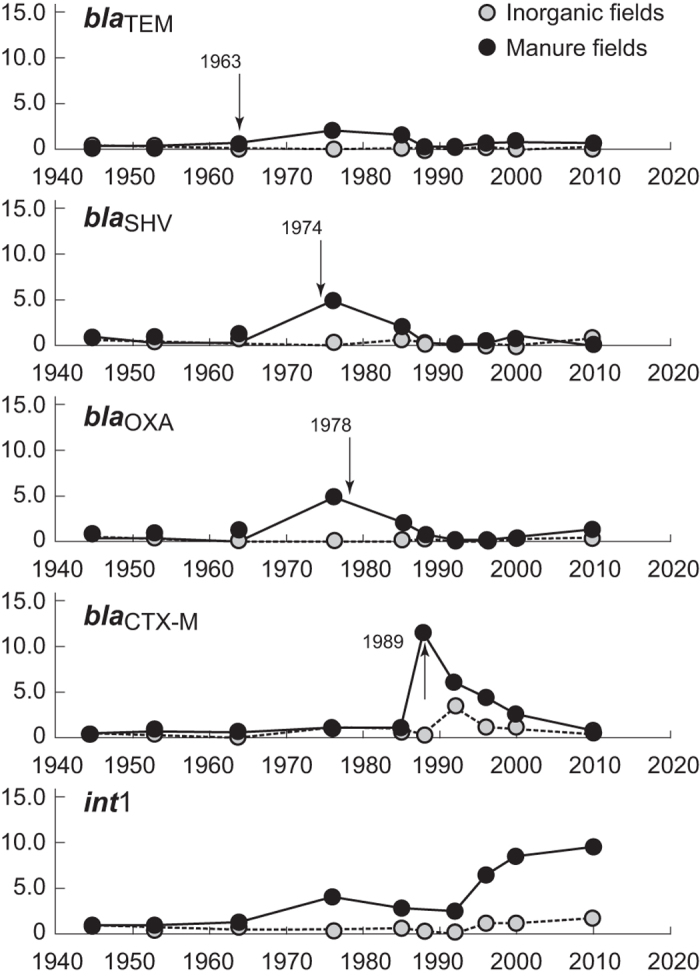
Relative abundance of *bla*_TEM_, *bla*_SHV_, *bla*_OXA_, *bla*_CTX-M_ and *int*1 gene levels in post-1940 archived soils from fields that have only received manure (M) or inorganic fertiliser (IF) applications since 1894. Data are reported as ratios of gene abundances (normalised to 16S-rRNA bacterial levels) in each sample relative to background levels determined from 1923 and 1938 samples. Dashed arrows correspond to dates when each gene was first reported in the literature associated with clinical isolates. Each point typically represents the mean of duplicate analyses for soil archive sample.

Mean gene-abundances in soils from before 1940 were as follows (log transformed values; see Tables S1 and S2): 16S rRNA = 9.50 ± 0.08 (±standard deviation), *bla*_TEM_ = 5.09 ± 0.11, *bla*_SHV_ = 4.27 ± 0.22, *bla*_OXA_ = 4.33 ± 0.44, *bla*_CTX-M_ = 4.61 ± 0.17, and *int*1 = 3.97 ± 0.24 gene copies g^-1^-soil dry-weight. From these data and data presented in Tables S1 and S2 (see Supporting Information; SI), relative abundances of *bla*_TEM_, *bla*_SHV_, *bla*_OXA_, *bla*_CTX-M_ and *int*1 in post-1940 soils were determined and summarised in Fig. 1. As it can be seen, no major differences in relative abundances of the four ARGs were apparent in either M or IF soils prior to about 1960. However, *bla*_TEM_, *bla*_SHV_, and *bla*_OXA_ levels all increased in M soils in 1976 samples, cresting in the mid-1980s, and then declining thereafter. No equivalent increase in ARG levels was seen in IF soils, implying detected increases were specifically associated with long-term manure treatment. Similarly, *bla*_CTX-M_ levels did not vary over time in either M or IF soils prior to the 1980s, but then increased by almost 15× in M soils in 1988 samples and then progressively declined until 2010. Clearly, manure additions resulted in higher soil ARG levels of all four genes, but increases differed over time among genes. Interestingly, temporal levels of *int*1 genes in the soils differed somewhat from β-lactam ARGs, with a shallow increase occurring in parallel with increases in *bla*_TEM_, *bla*_SHV_, and *bla*_OXA_ levels in the 1970 and 1980s, but then greatly increasing from the early 1990s through 2010.

Four key questions arise from these data: do ARG levels significantly differ between M and IF soils; have ARGs significantly changed in “background” soils over time (i.e., IF; soil without manure addition); why do ARG patterns differ among genes in the M soils; and has reduced non-therapeutic antibiotic use in Danish agriculture changed soil ARG levels? To address the first question, normalised levels of *bla*_TEM_, *bla*_SHV_, *bla*_OXA_ and *bla*_CTX-M_ were combined for all IF and M samples post-1940 and statistically compared.

**Figure 2:**
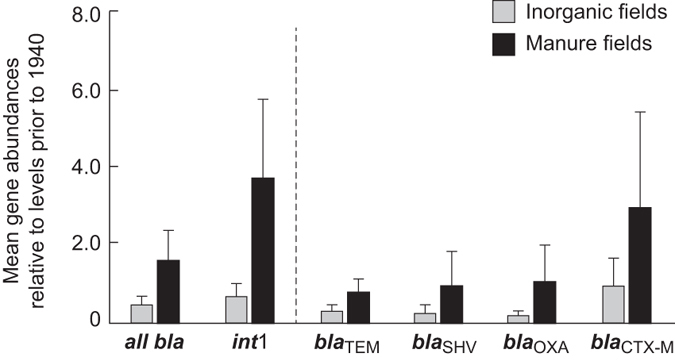
Mean relative abundances of the sum of all four *bla* genes, *int*1, *bla*_TEM_, *bla*_SHV_, *bla*_OXA_, and *bla*_CTX-M_ in post-1940 archived soils that have received continue manure (M) or inorganic fertiliser (IF) application since 1894. Data are mean ratios of each gene indicator (normalised to 16S-rRNA bacterial levels) in each sample relative to levels determined from 1923 and 1938 samples (n = 9 or 10). Error bars refer to 95% confidence levels in the means.

Mean “total *bla* levels” were found to be significantly higher in M than in IF soils (paired t-test; p = 0.001; see Fig. 2). Furthermore, individual *bla* gene and *int*1 levels were always greater in M versus IF soils, although differences were not always statistically significant (Wilcoxon Ranked Sum test; <0.05). Statistical comparisons were M > IF for *bla*_TEM_, p = 0.092; *bla*_SHV_, p = 0.051; *bla*_OXA_, p = 0.005; *bla*_CTX-M_, p = 0.037; and *int*1, p = 0.005. Despite these trends among ARGs, the greatest difference in detected gene abundances between M and IF soils was observed for *int*1 (Fig. 2), although this is not necessarily surprising given that *int1* is not specific to β-lactam ARGs, and may be more associated with other factors, such as continued manure application onto the fields.

The second question is more general and relates to changes in ARG levels in M versus IF soils since 1923. This question is best answered using log-transformed mean abundance data behind Fig. 2 (i.e., Tables S1 and S2). If one statistically compares normalised ARG levels (to 16S-rRNA gene abundances) before and after 1940, no significant long-term change in ARGs was apparent in the IF soils (paired t-test; p = 0.912), whereas ARG levels were much higher after 1940 in the M soils, albeit on the fringe of significance (p = 0.063). These data confirm no long-term change in soil β-lactam ARG levels were seen over the last century unless manure was applied. Our data suggest manure use for 100 years has approximately doubled ARG abundances in Lermarken M soils, increasing the probably of broader ARG exposure in drainage water and fodder crops grown in the soils^37^. However, data also show *int*1 levels increased by 10× in the M soils since the 1920s, which implies manure application has increased the intrinsic potential of these soils for horizontal gene transfer (HGT). Although it is speculation, we suspect continued manure use (and resultant increased bacterial permissiveness^38^) may be explain why *int*1 levels have increased since the mid-1990s despite the Danish ban on non-therapeutic antibiotic use in agriculture. ARG levels would appear to have declined in the manure, but the manure still contains *int*1 that continues to be released in the soils.

These observations are consistent with previous observations of the impact of manure and wastewater applications on ARG and *int*1 levels in agricultural fields^39^, although the impact of 100 years of manure applications has not been reported. Jechalke *et al.*^40^ used chronosequence data from different Mexican fields irrigated with wastewater for up to 100 years, and found higher AR and *int*1 abundances in fields irrigated the longest, although their study did not assess broader spectrum β-lactam ARGs. Further, Udikovic-Kolic *et al.*^41^ showed additions of manure increased ARG levels in resident antibiotic-resistant soil bacteria, although their study was relatively short-term. In contrast, we show that prolonged manure additions increase basal levels of β-lactam ARGs and *int*1 relative to IF applications, although dominant ARGs vary over time (Fig. 1). This implies ARG levels detected at any moment are dominated by more immediate factors, such as prevalent ARGs in “contemporary” manure and other factors that might influence ARG presence or survival in soils^42^.

**Figure 3:**
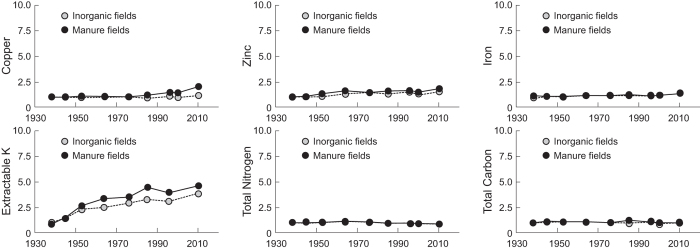
Relative mass of selected heavy metal and nutrients in archived soils compared with soils from before 1940 from manure (M) and inorganic fertiliser (IF) fields. Specific metals or nutrients are noted on y-axes and reported as ratios (i.e., mass of metal per date/mean mass of metal in samples from 1923 and 1938 samples).

The third and fourth questions are related to factors that might explain the appearance and disappearance patterns of *bla*_TEM_, *bla*_SHV_, *bla*_OXA_ and *bla*_CTX-M_ in the soils. Clearly, co-selection by heavy metals or nutrients in the manure or soil, and/or sub-therapeutic antibiotic exposures are options^25^,^42^,^43^. However, if one examines metal and soil nutrient levels over time (see Figs 3 and S1, and Tables S3 and S4), different temporal patterns are apparent for metals and nutrients compared with β-lactam ARGs. Some bivariate correlations were seen between selected ARGs and soil parameters (see Table S5), but no overarching trends were observed, except isolated correlations in selected M versus IF soils. In fact, with the exception of increasing Cu levels in M soils after 1990, and Zn, P and K levels for longer periods, M and IF soils were historically very similar (see Figs 3 and S1). Temporary increases in Ni, Cr and Hg were seen for short windows in the M soils (see Figure S1), possibly related to co-contamination in the manure with metals from other sources, but these metals did not correlate with detected *bla* gene or *int*1 levels, except on an incidental basis. No antibiotics analyses were performed here because such results would be meaningless given long storage time of the archived soils^44^. Therefore, other explanations are needed for ARG patterns over time.

Although various unknown factors might explain our patterns, we hypothesised observed β-lactam ARG patterns over time might reflect influences of observed AR across broader society, such as antibiotic use across sectors. Unfortunately, until the 1990s when DANMAP was initiated^34^, little is publically known about absolute antibiotic use in agriculture. Broad and/or extended spectrum β-lactam antibiotics have been used in Denmark since the late 1950s, including agriculture, although anecdotal evidence hint early use of β-lactam antibiotics (e.g., penicillins, cephalosporins, oxyimino β-lactams, etc.)^45^ was primarily in medicine rather than agriculture. Therefore, to understand ARG patterns in our soils, one must better understand contemporary ARGs being detected in parallel clinical settings.

To test this hypothesis, the literature was surveyed to determine when each β-lactam ARG was first seen in clinical isolates, which is annotated on Fig. 1. As can be seen, the first clinical appearance of the four *bla* genes assessed here matches appearance data in our soil series. Literature data show the genes sequentially appeared in clinical isolates as follows: *bla*_TEM_ (1963), *bla*_SHV_ (1974), *bla*_OXA_ (1978) and *bla*_CTX-M_ (1989)^18^,^19^,^20^,^21^. Although these data are not specific to Denmark, they indicate rough appearance of these ARGs across the developed world because first isolation dates include European examples^45^,^46^. Therefore, we suggest detected ARGs in the archived soils reflect a broader expansion of these ARGs across society, implying clinical and agricultural antibiotic and resistance development are mutually influential.

Interestingly, the appearance of each gene over time in the soil samples (i.e., when it first became elevated relative to baseline) is quite consistent with the evolution of resistance development within a medical context. *bla*_TEM_ and *bla*_SHV_ code for structurally similar β-lactamases^45^ and were the dominant form of β-lactam resistance detected prior to 1979^47^. However, as newer antibiotics were developed and employed, the prevalence of OXA-type β-lactamases increased (e.g., coded by *bla*_OXA_), and slow substitution of OXA β-lactams occurred in AR strains, possibly because they confer extended spectrum resistance and provide an ecological advantage over strains with *bla*_TEM_ or *bla*_SHV_^48^. This does not mean *bla*_TEM_ and *bla*_SHV_ β-lactamases disappeared because different phenotypes continued to be identified^47^,^49^, but their association with resistant infections declined. However, in the late 1980s, CTX-M β-lactamases appeared which are structurally different from previous β-lactam enzymes, have broader spectrum, and have since come to dominate over most other β-lactam enzymes since the late 1990s. This expansion is possibly due to accelerated evolution, movement onto promiscuous plasmids, and-or dispersion of *bla*_CTX-M_ genes across clinical and environmental settings^46^. For example, the emergence of CTX-M β-lactamases was simultaneously seen in Europe and South America^21^ and believed to have migrated on mobile genetic elements from environmental *Kluyvera* strains to organisms of clinical importance^50^,^51^.

Therefore, if manure-borne *bla* genes in archived soils and parallel clinical resistance are roughly synchronous, the question is which came first: environmental or clinical *bla* AR. Based on our data, one cannot say. However, if one considers the connectedness of environmental and human-associated AR, including cross-dissemination pathways via exposed water, food (vegetables, milk, meat, etc.) and even humans at the interface between agriculture and clinics, cross-dissemination is probable. In reality, our data suggest such a debate is now moot because these genes have become pervasive across nature. Most importantly, this connectivity suggests one cannot address broader problems of increasing AR by employing only medical, agricultural or environmental solutions because acquired AR (regardless of where it emerges) readily migrates across sectors. Therefore, having an antibiotic treatment fail in hospital may have nothing to do with the acquisition of AR in the hospital.

Despite these pessimistic observations, our results provide room for optimism. Although each *bla* gene increased in the archived M soils, they all subsequently declined to near baseline levels by 2010, presumably because causes of *bla* genes being present in the manure declined, possibly due to reduced non-therapeutic use in Danish agriculture, which other AR metrics have shown^34^,^36^. Therefore, our data imply reducing non-therapeutic antibiotic use can reduce some environmental AR legacies and an environment can recover given time. However, our data suggest recovery is not universal because *int*1 genes have increased over the same period. This suggests *int*1 accumulation may be more associated with manure use on soils rather than specific antibiotic use. Interestingly, we see an increase in Cu levels in the M soils (see Fig. 3), which inversely parallels the decrease in *bla*_CTX-M_ gene levels, but also significantly correlates with increasing in *int*1 levels in the M soils (r = 0.673, p = 0.033; see Table S5). These data imply substitution of Cu and possibly Zn has occurred in Danish agriculture, which was recently admitted in new reporting data^34^. However, it also hints that substitution to Cu/Zn (in lieu of antibiotics) may be responsible for increasing *int*1 levels in the M fields, although this is not yet proven. Regardless, the value of such corroboration in our results is profound because it confirms that soil archives can tell important contemporary stories and also foretell cause-and-effects that would not be apparent otherwise.

This study provides both optimistic and concerning results. A strong bridge between clinical and agricultural AR is apparent and to reduce globally increasing AR, antibiotic use and stewardship must improve across all sectors. If this is not done, AR from imprudent sectors will cross-contaminate the whole system. Optimistically, we show if one reduces the apparent pressure of antibiotic use in the environment, AR can be reduced (Fig. 1). A good starting point is the elimination of non-therapeutic antibiotic use in agriculture, which Denmark has done. However, recent increases in *int*1 and metal levels in the archived soils are concerning, and hint we may be solving one problem (reduced antibiotic use) by unintentionally creating another (increasing metals). Reducing non-therapeutic antibiotic use in agriculture is an important step, but one must consider the impact of alternate stewardship options within and across sectors or we will get nowhere relative to reducing AR in the future.

## Additional Information

**How to cite this article**: Graham, D. W. *et al.* Appearance of β-lactam Resistance Genes in Agricultural Soils and Clinical Isolates over the 20^th^ Century. *Sci. Rep.* **6**, 21550; doi: 10.1038/srep21550 (2016).

## Supplementary Material

Supplementary Information
