# Supplementary material for: Appearance of β-lactam Resistance Genes in Agricultural Soils and Clinical Isolates over the 20th Century
Source: Sci Rep. 2016 Feb 16;6:21550. doi: 10.1038/srep21550 (PMC4754643; doi:10.1038/srep21550)
Supplement: Supplementary Information [file srep21550-s1.pdf]

Supporting Information

Appearance of β-lactam Resistance Genes in Agricultural Soils and Clinical Isolates over the 20th Century

David W. Graham[1*], Charles W. Knapp[2*], Bent T. Christensen[3],

Seánín McCluskey[2], Jan Dolfing[1]

[1] – School of Civil Engineering & Geosciences, Newcastle University, Newcastle upon Tyne, United Kingdom, NE1 7RU

[2] - Department of Civil and Environmental Engineering, University of Strathclyde, Glasgow, United Kingdom, G1 1XJ

[3] – Department of Agroecology, Aarhus University, AU-Foulum, DK-8830 Tjele, Denmark

Content: Five tables and one figure.

Corresponding authors: Prof David W. Graham
School of Civil Engineering & Geosciences
Newcastle University
Newcastle upon Tyne, United Kingdom NE1 7RU
E-mail: d.graham@ncl.ac.uk, Phone: +44 (0) 191 208 7930

Dr Charles Knapp
Department of Civil and Environmental Engineering
University of Strathclyde
Glasgow, United Kingdom G1 1XJ
E-mail: charles.knapp@strath.ac.uk, Phone: +44 (0)141 548 3351

Table S1. qPCR quantification of mean 16S-rRNA, broad spectrum β-lactam resistance, and integron integrase genes (log abundances/g dry-weight soil) detected in archived soils from fields only provided inorganic fertilisers (IF) since 1894. Means of duplicates are reported. Measured duplicate samples were always < 0.5 log of each other.

**Year**	16S-rRNA	bla_{TEM}	bla_{SHV}	bla_{OXA}	$bla_{CTX\text{-}M}$	*int*1
1923	9.44	5.00	3.99	4.68	4.77	3.78
1938	9.50	5.07	4.42	3.69	4.71	4.29
1945	9.49	4.91	4.10	3.98	3.37	3.80
1953	9.58	4.99	3.96	4.13	4.14	3.84
1964	9.74	4.66	3.41	3.97	3.43	4.11
1976	9.80	4.74	3.31	4.10	5.05	3.90
1985	9.40	4.34	4.16	3.67	4.66	3.74
1988	9.80	4.21	3.67	3.61	4.48	3.91
1992	9.89	5.21	3.59	3.69	5.67	3.80
1996	9.72	4.86	3.44	4.19	5.05	4.34
2000	10.0	5.00	3.10	4.36	5.27	4.60
2010	9.81	5.07	4.47	4.51	4.66	4.56
**Mean**	**9.68**	**4.84**	**3.80**	**4.05**	**4.60**	**4.05**
**SD**	**0.20**	**0.30**	**0.45**	**0.35**	**0.68**	**0.32**
**95% CI**	**0.11**	**0.16**	**0.24**	**0.19**	**0.37**	**0.17**

Table S2. qPCR quantification of mean 16S-rRNA, broad spectrum β-lactam resistance, and integron intergase genes (log abundances/g dry weight soil) detected in archived soils from fields provided only manure (M) since 1894. Means of duplicates are reported. Measured duplicate samples were always < 0.5 log of each other.

**Year**	16S-rRNA	bla_{TEM}	bla_{SHV}	bla_{OXA}	bla_{CTX-M}	*int*1
1923	9.44	5.24	4.20	4.55	4.57	3.80
1938	9.62	5.04	4.46	4.4	4.38	4.01
1945	9.54	4.91	4.23	4.26	4.25	4.03
1953	9.63	4.88	4.11	4.34	4.67	4.05
1964	9.62	5.05	3.98	3.90	4.52	4.21
1976	9.14	5.10	4.63	4.83	4.32	4.25
1985	9.21	5.06	4.34	4.53	4.28	4.21
1988	10.4	5.55	4.24	5.20	6.65	N/A[a]
1992	9.92	5.21	4.13	4.20	5.91	4.84
1996	9.40	4.94	3.74	4.00	5.25	4.73
2000	9.56	5.14	4.22	4.27	5.17	5.00
2010	9.76	5.10	3.97	4.90	4.60	5.26
**Mean**	**9.60**	**5.10**	**4.19**	**4.45**	**4.88**	**4.41**
**SD**	**0.32**	**0.18**	**0.23**	**0.38**	**0.74**	**0.46**
**95% CI**	**0.18**	**0.10**	**0.13**	**0.21**	**0.40**	**0.25**

Notes: [a] N/A = Not available.

Table S3. Metal and nutrient concentrations in archived soils from fields provided inorganic fertilisers (IF) since 1894. Nutrient levels were determined at the time of historic sampling, whereas heavy metals were analysed on archived soils during this study.

**Year**	As (ppm)[a]	Cd (ppm)	Co (ppm)	Cr (ppm)	Cu (ppm)	Hg (ppb)	Ni (ppm)	Pb (ppm)	Zn (ppm)	TC (g C/100 g)	TN (g N/100 g)	Ext P (mg P/100 g)	Ext K (mg K/100 g)
1923	3.3	0.15	2.6	6.2	5.76	43	3.6	12.1	17.3	0.118	1.44	6.3	2.4
1938	4.0	0.16	2.4	5.9	4.20	63	3.2	12.3	15.8	0.111	1.49	3.9	4.3
1945	3.9	0.17	2.3	6.2	4.09	66	3.2	12.8	16.9	0.117	1.36	7.8	5.2
1953	3.9	0.19	2.6	5.8	3.67	60	2.9	12.5	15.7	0.115	1.36	12.3	3.6
1964	4.3	0.19	2.5	6.4	4.04	107	3.4	12.2	21.6	0.125	1.38	13.2	5.3
1976	4.0	0.20	2.9	6.7	4.18	74	3.6	13.1	21.0	0.108	1.46	15.6	8.3
1985	4.1	0.20	3.2	12.7	3.85	61	6.1	12.7	20.0	0.106	1.25	17.4	5.9
1996	3.9	0.23	2.5	9.5	4.31	73	4.9	14.2	23.7	0.12	1.31	16.2	6.9
2000	3.6	0.20	2.9	14.8	4.03	49	7.4	12.3	20.0	0.102	1.24	N/A[b]	5.4
2010	4.2	0.19	3.1	8.0	4.63	56	3.7	13.6	24.2	0.106	1.19	20.4	7.1
**Mean**	**3.92**	**0.188**	**2.70**	**8.22**	**4.28**	**65.2**	**4.20**	**12.8**	**19.6**	**0.113**	**1.35**	**12.6**	**5.44**
**SD**	**0.290**	**0.023**	**0.31**	**3.16**	**0.58**	**17.6**	**1.48**	**0.7**	**3.10**	**0.007**	**0.10**	**5.53**	**1.74**
**95% CI**	**0.180**	**0.014**	**0.19**	**1.96**	**0.36**	**10.9**	**0.92**	**0.4**	**1.92**	**0.005**	**0.06**	**3.43**	**1.08**

Notes: [a] Mass per mass of dry soil

[b] N/A = Not available.

Table S4. Metal and nutrient concentrations in archived soils from manure (M) fields since 1894. Nutrient levels were determined at the time of historic sampling, whereas heavy metals were analysed on archived soils during this study.

**Year**	As (ppm)[a]	Cd (ppm)	Co (ppm)	Cr (ppm)	Cu (ppm)	Hg (ppb)	Ni (ppm)	Pb (ppm)	Zn (ppm)	TC (g C/100 g)	TN (g N/100 g)	Ext P (mg P/100 g)	Ext K (mg K/100 g)
1923	1.7	0.11	2.1	5.0	4.38	27	2.7	9.9	13.5	0.133	1.53	4.2	1.4
1938	2.4	0.14	2.5	5.6	4.54	78	3.1	12.0	15.9	0.126	1.68	2.7	3
1945	2.9	0.14	2.4	5.6	4.18	59	3.1	12.0	17.6	0.131	1.5	6	5.7
1953	2.7	0.16	2.4	5.9	4.41	80	3.2	12.7	18.4	0.133	1.54	10.8	4.4
1964	2.9	0.16	2.5	6.2	4.21	130	3.2	13.3	23.6	0.128	1.6	13.8	4.2
1976	3.1	0.18	2.7	7.6	4.30	54	4.2	12.6	21.3	0.115	1.6	14.4	6.5
1985	3.4	0.19	4.2	22.5	4.63	65	10.6	14.4	23.4	0.122	1.37	18.3	7.5
1996	3.1	0.21	3.1	15.9	6.08	57	8.3	14.0	25.1	0.135	1.42	16.2	8.1
2000	3.0	0.18	2.6	11.8	6.23	46	5.8	12.8	22.1	0.122	1.38	N/A[b]	5.5
2010	3.4	0.18	3.5	7.8	9.16	53	4.4	13.5	28.3	0.127	1.31	19.2	7.8
**Mean**	**2.86**	**0.165**	**2.80**	**9.39**	**5.21**	**64.9**	**4.86**	**12.7**	**20.9**	**0.127**	**1.49**	**11.7**	**5.41**
**SD**	**0.51**	**0.029**	**0.63**	**5.74**	**1.58**	**27.5**	**2.64**	**1.27**	**4.53**	**0.006**	**0.12**	**6.15**	**2.19**
**95% CI**	**0.31**	**0.018**	**0.39**	**3.56**	**0.98**	**17.0**	**1.64**	**0.79**	**2.81**	**0.004**	**0.07**	**3.81**	**1.35**

Notes: [a] Mass per mass of dry soil

[b] N/A = Not available.

Figure S1. Relative mass of selected heavy metal and nutrients in archived soils compared with soils from before 1940 from manure (M) and inorganic fertiliser (IF) fields. Specific metals or nutrients are noted on y-axes and reported as ratios (i.e., mass of metal per date/mean mass of metal in samples from 1923 and 1938 samples).

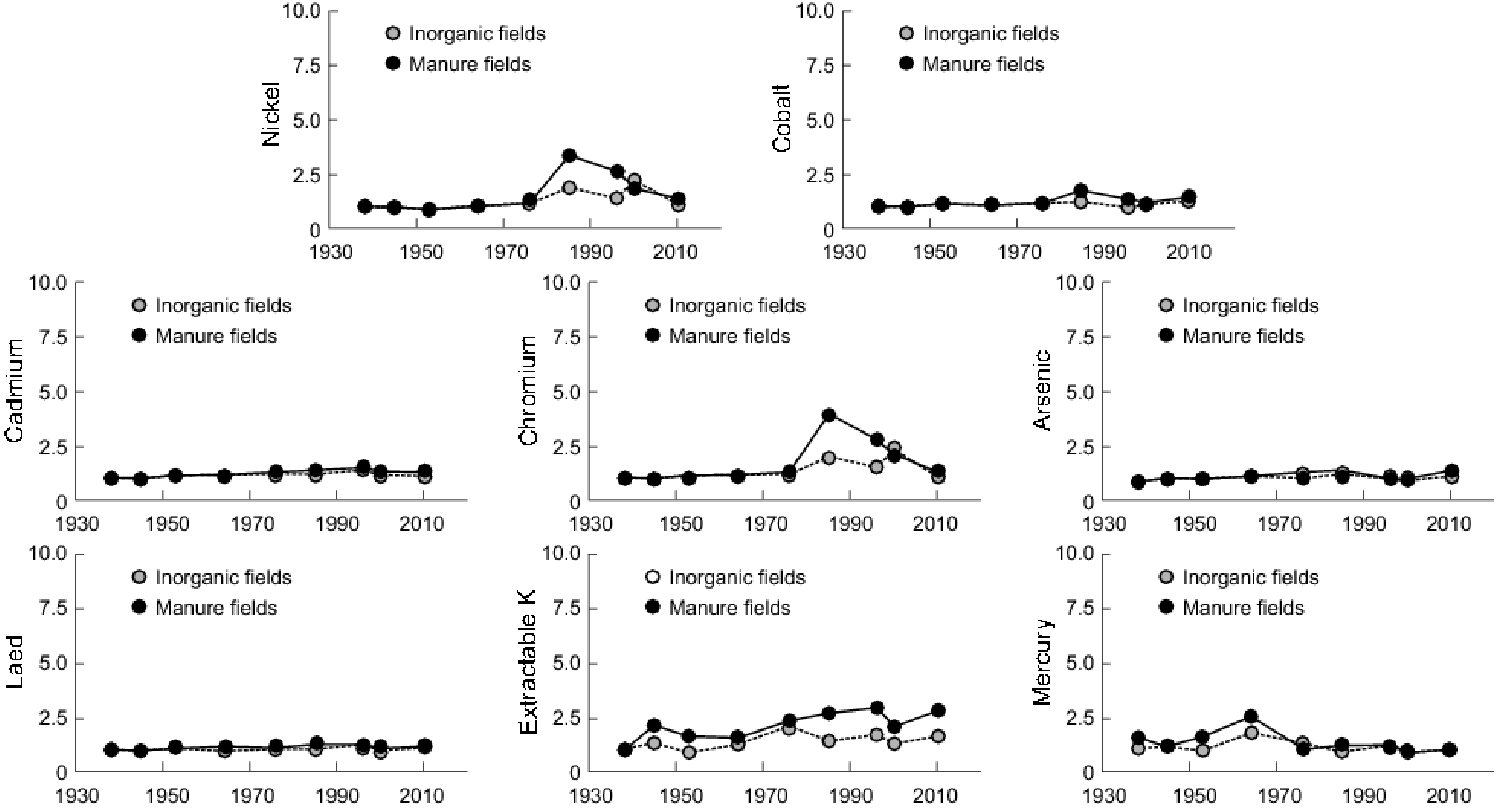

Table S5. Spearman's rank correlation coefficents between time, metals, nutrients and genes versus time or specific ARGs or *int*1 genes in archived soils from manure (M) and inorganic (IF) fertilised fields after 1940. Significant correlations are in bold and p-values in brackets.

	**Inorganic**						**Manure**					
	Time[a]	bla_{TEM}	bla_{SHV}	bla_{OXA}	$bla_{CTX\text{-}M}$	*int*1	Time	bla_{TEM}	bla_{SHV}	bla_{OXA}	$bla_{CTX\text{-}M}$	*int*1
Time	-	-0.146<br>(0.688)	-0.127<br>(0.726)	0.018<br>(0.960)	**0.673**[b]<br>(0.033)	0.467<br>(0.174)	-	0.055<br>(0.881)	-0.491<br>(0.150)	-0.012<br>(0.973)	0.418<br>(0.229)	**0.733**[b]<br>(0.016)
Mo	0.665 [b]<br>(0.036)	-0.07<br>(0.847)	0.238<br>(0.508)	0.043<br>(0.907)	0.616<br>(0.058)	0.427<br>(0.219)	**0.875**[c]<br>(0.001)	-0.219<br>(0.544)	-0.669[b]<br>(0.035)	-0.291<br>(0.415)	0.474<br>(0.166)	0.505<br>(0.137)
Cu	-0.103<br>(0.777)	0.438<br>(0.206)	0.624<br>(0.054)	**0.661**[b]<br>(0.038)	-0.455<br>(0.187)	0.515<br>(0.128)	**0.709**[b]<br>(0.022)	-0.055<br>(0.881)	-0.273<br>(0.446)	-0.073<br>(0.841)	0.103<br>(0.777)	**0.673**[b]<br>(0.033)
Pb	0.539<br>(0.108)	-0.292<br>(0.413)	-0.115<br>(0.751)	-0.055<br>(0.881)	0.164<br>0.651)	0.285<br>(0.425)	**0.806**[c]<br>(0.005)	0.176<br>(0.627)	-0.321<br>(0.365)	-0.110<br>(0.763)	0.491<br>(0.150)	**0.648**[b]<br>(0.043)
Zn	**0.736**[b]<br>(0.015)	-0.055<br>(0.880)	0.474<br>(0.166)	0.109<br>(0.763)	0.419<br>(0.228)	0.584<br>(0.077)	**0.879**[c]<br>(0.001)	0.164<br>(0.651)	-0.382<br>(0.276)	0.104<br>(0.776)	0.321<br>(0.365)	**0.709**[b]<br>(0.022)
Ni	**0.726**[b]<br>(0.018)	0.269<br>(0.452)	-0.116<br>(0.750)	-0.018<br>(0.960)	0.628<br>(0.052)	0.317<br>(0.372)	**0.872**[c]<br>(0.001)	-0.012<br>(0.973)	-0.482<br>(0.159)	-0.245<br>(0.494)	**0.701**[b]<br>(0.024)	0.537<br>(0.110)
Co	0.612<br>(0.060)	0.160<br>(0.660)	-0.055<br>(0.880)	-0.141<br>(0.698)	0.483<br>(0.157)	-0.171<br>(0.636)	**0.823**[c]<br>(0.003)	-0.378<br>(0.281)	**-0.738**[b]<br>(0.015)	-0.245<br>(0.494)	0.555<br>(0.096)	0.354<br>(0.316)
Mn	0.236<br>(0.511)	0.146<br>(0.688)	0.515<br>(0.128)	0.564<br>(0.090)	-0.103<br>(0.777)	0.261<br>(0.467)	**0.891**[c]<br>(0.001)	-0.200<br>(0.580)	**-0.648**[b]<br>(0.043)	0.024<br>(0.947)	0.527<br>(0.117)	0.479<br>(0.162)
Fe	0.426<br>(0.220)	0.409<br>(0.241)	0.462<br>(0.179)	0.176<br>(0.626)	0.274<br>(0.444)	0.340<br>(0.336)	**0.791**[c]<br>(0.006)	-0.423<br>(0.223)	**-0.779**[c]<br>(0.008)	-0.191<br>(0.596)	0.423<br>(0.223)	0.337<br>(0.340)
As	0.302<br>(0.397)	-0.117<br>(0.747)	0.554<br>(0.097)	-0.160<br>(0.659)	0.246<br>(0.493)	-0.074<br>(0.839)	**0.869**[c]<br>(0.001)	-0.263<br>(0.463)	**-0.765**[b]<br>(0.010)	-0.222<br>(0.538)	0.514<br>(0.129)	0.422<br>(0.224)

	Time	bla_{TEM}	bla_{SHV}	bla_{OXA}	bla_{CTX-M}	*int*1	Time	bla_{TEM}	bla_{SHV}	bla_{OXA}	bla_{CTX-M}	*int*1
Cd	**0.777**[c]<br>(0.008)	-0.330<br>(0.351)	-0.354<br>(0.315)	-0.379<br>(0.280)	0.801[c]<br>(0.005)	0.093<br>(0.798)	**0.858**[c]<br>(0.001)	-0.049<br>(0.892)	-0.549<br>(0.100)	-0.199<br>(0.582)	**0.735**[b]<br>(0.016)	0.482<br>(0.159)
Ca	**-0.752**[b]<br>(0.012)	0.294<br>(0.409)	-0.141<br>(0.698)	-0.128<br>(0.724)	-0.471<br>(0.169)	-0.343<br>(0.333)	-0.566<br>(0.088)	-0.043<br>(0.906)	0.265<br>(0.460)	-0.650[b]<br>(0.042)	-0.271<br>(0.449)	-0.357<br>(0.311)
P	0.278<br>(0.437)	-0.257<br>(0.474)	0.210<br>(0.561)	0.265<br>(0.458)	-0.123<br>(0.734)	0.395<br>(0.258)	**0.801**[c]<br>(0.005)	0.217<br>(0.546)	-0.304<br>(0.392)	-0.281<br>(0.431)	0.255<br>(0.478)	**0.789**[c]<br>(0.007)
Cr	**0.821**[c]<br>(0.004)	0.043<br>(0.907)	-0.158<br>(0.663)	-0.170<br>(0.638)	**0.681**[b]<br>(0.030)	0.347<br>(0.327)	**0.875**[c]<br>(0.001)	-0.018<br>(0.960)	-0.486<br>(0.154)	-0.251<br>(0.485)	**0.693**[b]<br>(0.026)	0.529<br>(0.116)
Mg	-0.097<br>(0.790)	0.463<br>(0.178)	-0.055<br>(0.879)	0.354<br>(0.316)	0.014<br>(0.970)	0.139<br>(0.702)	**0.782**[c]<br>(0.008)	-0.216<br>(0.549)	**-0.667**[b]<br>(0.035)	0.019<br>(0.958)	0.578<br>(0.080)	0.292<br>(0.412)
Ba	-0.311<br>(0.382)	-0.049<br>(0.893)	-0.207<br>(0.565)	0.128<br>(0.724)	-0.384<br>(0.273)	0.091<br>(0.802)	0.573<br>(0.083)	0.218<br>(0.545)	-0.187<br>(0.605)	-0.232<br>(0.519)	0.237<br>(0.510)	**0.679**[b]<br>(0.031)
Al	0.049<br>(0.893)	-0.086<br>(0.812)	0.351<br>(0.320)	0.326<br>(0.358)	-0.351<br>(0.320)	0.535<br>(0.111)	**0.765**[c]<br>(0.010)	0.144<br>(0.691)	-0.420<br>(0.227)	-0.129<br>(0.722)	0.401<br>(0.250)	0.609<br>(0.062)
Na	**-0.790**[c]<br>(0.007)	-0.053<br>(0.885)	-0.117<br>(0.747)	0.136<br>(0.708)	-0.599<br>(0.067)	-0.420<br>(0.227)	-0.354<br>(0.316)	0.028<br>(0.939)	0.402<br>(0.249)	0.101<br>(0.781)	-0.451<br>(0.191)	-0.132<br>(0.717)
K	0.497<br>(0.143)	-0.321<br>(0.366)	0.569<br>(0.086)	0.071<br>(0.845)	0.213<br>(0.554)	0.426<br>(0.219)	**0.646**[b]<br>(0.044)	-0.190<br>(0.599)	-0.570<br>(0.086)	-0.306<br>(0.390)	0.190<br>(0.599)	0.494<br>(0.147)
Hg	-0.018<br>(0.960)	-0.578<br>(0.080)	0.236<br>(0.511)	-0.467<br>(0.174)	0.091<br>(0.803)	-0.079<br>(0.829)	-0.248<br>(0.489)	0.273<br>(0.446)	0.345<br>(0.328)	0.183<br>(0.613)	0.188<br>(0.603)	-0.115<br>(0.751)
TC	-0.511<br>(0.132)	-0.085<br>(0.815)	0.389<br>(0.266)	0.024<br>(0.947)	-0.195<br>(0.590)	0.085<br>(0.815)	-0.305<br>(0.392)	0.579<br>(0.079)	0.482<br>(0.159)	0.098<br>(0.787)	-0.439<br>(0.204)	0.250<br>(0.486)
TN	**-0.802**[c]<br>(0.005)	-0.046<br>(0.900)	0.316<br>(0.374)	-0.067<br>(0.854)	-0.559<br>(0.093)	-0.474<br>(0.166)	**0.693**[b]<br>(0.026)	-0.067<br>(0.854)	0.371<br>(0.291)	0.349<br>(0.324)	0.012<br>(0.973)	**-0.693**[b]<br>(0.026)

	Time	*bla*$_{TEM}$	*bla*$_{SHV}$	*bla*$_{OXA}$	*bla*$_{CTX-M}$	*int*1	Time	*bla*$_{TEM}$	*bla*$_{SHV}$	*bla*$_{OXA}$	*bla*$_{CTX-M}$	*int*1
ExtP	**0.967**[c] (0.001)	-0.176 (0.651)	0.067 (0.865)	-0.183 (0.637	**0.683**[b] (0.042)	0.183 (0.637)	**0.967**[c] (0.001)	-0.067 (0.865)	-0.633 (0.067)	0.025 (0.949)	0.400 (0.286)	0.600 (0.088)
ExtK	**0.806**[c] (0.005)	-0.316 (0.374)	0.091 (0.803)	-0.200 (0.580)	0.418 (0.229)	0.285 (0.425)	**0.782**[c] (0.008)	-0.164 0.651)	**-0.745**[b] (0.013)	-0.317 (0.372)	0.467 (0.174)	0.442 (0.200)
bla$_{TEM}$	-0.146 (0.688)	-	0.298 (0.403)	0.584 (0.077)	0.061 (0.868)	0.006 (0.987)	0.055 (0.881)	-	**0.758**[b] (0.011)	0.329 (0.353)	-0.115 (0.751)	0.588 (0.074)
bla$_{SHV}$	-0.127 (0.726)	0.298 (0.403)	-	0.370 (0.293)	-0.224 (0.533)	0.188 (0.603)	-0.491 (0.150)	**0.758**[b] (0.011)	-	0.390 (0.265)	-0.345 (0.328)	0.103 (0.777)
bla$_{OXA}$	0.018 (0.960)	0.584 (0.077)	0.370 (0.293)	-	-0.236 (0.511)	0.564 (0.090)	-0.012 (0.973)	0.329 (0.353)	0.390 (0.265)	-	-0.171 (0.637)	0.165 (0.649)
bla$_{CTX-M}$	**0.673**[b] (0.033)	0.061 (0.868)	-0.224 (0.533)	-0.236 (0.511)	-	-0.030 (0.934)	0.418 (0.229)	-0.115 (0.751)	-0.345 (0.328)	-0.171 (0.637)	-	-0.067 (0.855)
*int*1	0.467 (0.174)	0.006 (0.987)	0.188 (0.603)	0.564 (0.090)	-0.030 (0.934)	-	**0.733**[b] (0.016)	0.588 (0.074)	0.103 (0.777)	0.165 (0.649)	-0.067 (0.855)	-

Notes: [a] Nutrients and metals = mass per mass of dry soil; genes = copies per mass of dry soil; time = years

[b] 95% Confidence correlation

[c] 99% Confidence correlation
